# Novel Hybrid of Typical Enteropathogenic *Escherichia coli* and Shiga-Toxin-Producing *E. coli* (tEPEC/STEC) Emerging From Pet Birds

**DOI:** 10.3389/fmicb.2018.02975

**Published:** 2018-12-06

**Authors:** Rosely Martins Gioia-Di Chiacchio, Marcos Paulo Vieira Cunha, Lilian Rose Marques de Sá, Yamê Minieiro Davies, Camila Bueno Pacheco Pereira, Fernando Henrique Martins, Danielle Dias Munhoz, Cecilia Mari Abe, Marcia Regina Franzolin, Luis Fernando dos Santos, Beatriz Ernestina Cabilio Guth, Waldir Pereira Elias, Roxane Maria Fontes Piazza, Terezinha Knöbl

**Affiliations:** ^1^Department of Pathology, School of Veterinary Medicine and Animal Science, São Paulo, Brazil; ^2^School of Veterinary Medicine, Paulista University, São Paulo, Brazil; ^3^Butantan Institute, São Paulo, Brazil; ^4^Adolfo Lutz Institute, São Paulo, Brazil; ^5^Universidade Federal de São Paulo, São Paulo, Brazil

**Keywords:** zoonosis, public health, animal diseases, psittacine birds, diarrheagenic *E. coli*

## Abstract

Exotic psittacine birds have been implicated as reservoir of diarrheagenic *Escherichia coli* (*E. coli*), including enteropathogenic *E. coli* (EPEC) and Shiga-toxin producing *E. coli* (STEC). Here, we present a genotypic and phenotypic characterization of typical EPEC/STEC hybrid strains isolated from exotic psittacine birds. The strains were positive for *eae*, *bfp*A, and s*tx*2f genes, belong to serotype O137:H6 and ST2678. Two strains were subject to whole genome sequencing, confirming the presence of the virulence factors of both *E. coli* pathotypes. Phenotypical *in vitro* tests confirmed their ability to adhere to HeLa cells and cause cytotoxicity to Vero cells. The rabbit ileal loop assays showed the attaching and effacing lesion, in addition to inflammatory process and overproduction of intestinal mucus. This is the first report of hybrid typical EPEC/STEC (O137:H6/ST2678) strains isolated from companion psittacine birds and the results suggest zoonotic risks.

## Introduction

*Escherichia coli* is one of the agents most frequently involved in the imbalance of young psittacine birds’ intestinal microbiota, which is exclusively composed by Gram-positive bacteria (Xenoulis et al., 2010). The colonization in psittacine birds by Gram-negative bacteria is an unwanted sanitary problem, which influenced by unsatisfactory farming conditions, may represent zoonotic risk (Gioia-Di Chiacchio et al., 2016).

Diarrheagenic *E. coli* (DEC) classification comprises distinct *E. coli* pathotypes based on different genetic virulence markers (Gomes et al., 2016). This classification is highly helpful in understanding the mechanisms implicated in the occurrence of diarrhea. However, the advance in laboratory and molecular techniques has revealed that the same serogroup may be present in more than one pathotype (Campos et al., 2004). Likewise, certain virulence factors are not confined to a single DEC category. Mobile elements can disseminate virulence genes from one lineage to another, thereby generating intermediary groups, with a combination of virulence types or hybrids (Nyholm et al., 2015). An example of this situation is the enteroaggregative *E. coli* heat-stable enterotoxin (EAST), initially described in enteroaggregative *E. coli* (EAEC), but that may also be detected in enterotoxigenic *E. coli* (ETEC) and enteropathogenic *E. coli* (EPEC) strains (Dulguer et al., 2003; Muniesa et al., 2012).

Shiga-toxin producing *E. coli* (STEC) is a worldwide spread pathogen associated with human diseases, mainly diarrhea, hemorrhagic colitis and hemolytic-uremic syndrome (HUS) (Friesema et al., 2014). Recently, a hybrid EAEC/Stx2a-toxin-producing STEC lineage belonging to the O104:H4 serotype and able to form *in vivo* biofilm due to the presence of characteristic EAEC virulence genes were identified as the causative agent of a massive diarrhea outbreak in Europe (Muniesa et al., 2012).

Hybrid isolates of STEC and ETEC from human and diverse animal species, harboring the plasmid p7v were recently described (Leonard et al., 2016). The sequencing identified the genes that encoded for fimbria K88 and toxins STa, STb, Stx1c, Stx2g, and Stx2e. The authors concluded that the presence of mobile elements might contribute for the emergence of hybrid lineages of STEC, with increased virulence potential (Leonard et al., 2016). Production animals may lodge hybrid lineages, as described previously (Johura et al., 2017). The authors identified a hybrid STEC/ETEC strain from ruminant isolates in Bangladesh, which were positive for STa and Stx1/Stx2 toxins, and that were heterogeneous upon pulsed-field gel electrophoresis (PFGE). To date, no hybrid *E. coli* lineages have been reported in pet animals. This study described, for the first time, a hybrid lineage of typical EPEC (tEPEC) and STEC, isolated from small-size psittacine birds raised as pets. In a previous evaluation, we investigated the frequency of DEC in 171 fecal samples of companion psittacine pet birds. Amongst them, 42 birds were colonized by *E. coli*, and eight (4.6%) were STEC-positive (Gioia-Di Chiacchio et al., 2016). These results led us to a further investigation of these strains, thus, using whole genome sequencing, we found that the strains possessed sequences of *bfp* plasmid, and were classified as a hybrid of tEPEC and STEC; thus, herein we provide a genotypic and phenotypic characterization of these hybrid strains.

## Materials and Methods

### Bacterial Strains

This study has been approved by the University of São Paulo Ethics Committee and authorized for scientific purposes (CEUA 7423290414/2016). The written informed consent was obtained from the owners of the birds that participate in this study. Eight DEC strains belonging to the Avian Disease Laboratory’s Culture Collection (School of Veterinary Medicine and Animal Science, University of São Paulo, Brazil) were evaluated. The strains were isolated from fecal samples of clinically healthy cockatiels (*Nymphicus hollandicus*) and budgerigars (*Melopsittacus undulatus*) domiciled in the city of São Paulo, Brazil (Gioia-Di Chiacchio et al., 2016). They were characterized as putative hybrids after screening and positivity upon PCR for *eae*, *bfpA*, and *stx*2 genes, according to the protocols described elsewhere (Tsen and Jian, 1998; Elias et al., 2002; Mora et al., 2011).

### Virulence Genotyping, MLST, and Serotype Determination

The DEC and ExPEC virulence factors as well as the seven Shiga toxin-2 variants were searched by polymerase chain reaction (PCR), using the specific primers sequences and methodology as described in Supplementary Material (Table S1). Basically, the PCR was performed in 25 μL of mixture (1× PCR buffer, 1.5 mM MgCl_2_, 0.2 mM of each deoxyribonucleotide, 10 pM of primers, 0.5 U of Taq DNA polymerase, autoclaved ultra-pure water, and 3 μL of DNA template.

MLST technique comprises the amplification and sequencing of seven *housekeeping* genes, following the protocol described (Wirth et al., 2006), using the typing scheme proposed by Enterobase at Warwick Medical School^1^ (Wirth et al., 2006).

The serotypes were identified using agglutination tests as described (Guineée et al., 1981), with O and H *E. coli* anti-sera produced by the *E. coli* Reference Laboratory at the Adolfo Lutz Institute – (São Paulo, Brazil) (Guineée et al., 1981).

### *In vitro* Adhesion Assay in HeLa Cells and Cytotoxicity Assay in Vero Cells

Adhesion assays were performed as described (Scaletsky et al., 1984), with slight modifications. Semi confluent HeLa cells (ATCC: CCL-2) monolayers were grown on coverslips inserted in 24-well plates. Bacterial strains statically grown in TSB at 37°C during 18 h were added to HeLa cells monolayers, following a dilution of 1:25 (∼3 × 10^7^ bacteria) in Dulbecco’s Modified Eagle Medium (DMEM) supplemented with 2% fetal bovine serum (FBS) and 1% D-mannose. The preparations were then incubated for 6 h at 37°C, washed with phosphate buffer saline (PBS), fixed with 4% methanol and stained with Giemsa. The EPEC prototype strain E2348/69 was used as a control for the localized-adherence (LA) pattern (Levine et al., 1978). Non-infected HeLa cells were also used as a control.

The cytotoxicity assay in Vero cells followed the methodology described (Martins et al., 2015), employing bacterial supernatants in Luria Bertani (LB) broth (Merck) in the presence of 5 ng/mL of ciprofloxacin (Sigma-Aldrich) (Rocha and Piazza, 2007). *E. coli* O157:H7 (EDL933) and *E. coli* DH5α supernatants were used as positive and negative controls, respectively (Wells et al., 1983). The same assays were performed in the presence of polyclonal Stx2 antibody diluted 1:400 to evaluate the neutralization of the cytotoxic effect (Rocha and Piazza, 2007). All assays were performed in triplicates.

### Ileal Loop Fluid Accumulation and Histologic Test in Rabbits

A laparotomy of a male New Zealand rabbit was performed, connecting five-centimeter ileum segments, separated by 3 cm inter-loops, followed by 1 mL of bacterial culture inoculation (standardized at a concentration of 1 × 10^6^ UFC/mL) (Bergdoll, 1988). The atypical EPEC BA320 strain (serotype O55:H7) was used as a positive control (Rocha et al., 2011), PBS and DH10-B as negative controls. After 12 h, the rabbit was euthanized and its intestinal loops were examined for the presence of fluid and mucus accumulation. Half-millimeter-long intestinal fragments were fixed in 10% formaldehyde, and subsequently, dehydrated, diaphanized, and included in paraffin. The blocks were sectioned in Leica RM2145 microtome (Leica Biosystems, Germany) and stained with Giemsa. The histologic sections were observed under light microscope.

### Scanning and Transmission Electron Microscopy (SEM and TEM)

Infected and non-infected tissue fragments of the intestinal loops were fixed in 2.5% glutaraldehyde (v/v) in 0.1 M phosphate buffer, washed with 0.1 M sodium cacodylate buffer, post-fixed with 1% osmium tetroxide (OsO_4_) (v/v) in the same buffer solution, and dehydrated through a graded series of ethanol (50, 75, 85, 95, and 100% solutions).

For scanning electron microscopy (SEM), preparations were subsequently dried by the critical point method, mounted onto SEM stubs, sputter coated with gold, and examined under SEM (Quanta 250, FEI Company, Netherlands), operating at 12.5 kV and working distance of 7 mm. For transmission electron microscopy (TEM), preparations were also dehydrated with propylene oxide, and gradually infiltrated and embedded in Araldite. After polymerization at 60°C for 24–48 h ultra-thin sections were obtained (Leica EM UC7 ultra-microtome, Leica Biosystems), placed onto Formvar coated 200 meshes copper grids and stained with 2% aqueous uranyl acetate and lead citrate solutions. Grids were then analyzed under TEM (Tecnai G2, FEI Company, Netherlands), operating at 60 kV.

### Plasmid Analyses

Plasmid profiles sizes were analyzed by DNA linearization with S1 nuclease followed by PFGE (S1-PFGE). The plasmid incompatibility group analyses were determined according to the PCR-based replicon-typing (PBRT) method (Carattoli et al., 2005).

### Whole Genome Sequencing and Analysis

Two strains isolated from different hosts (cockatiel and budgerigar) were selected for whole genome sequencing: CA14 and PA58. Total genomic DNA were extracted and used to construct paired-end libraries (150-base reads) sequenced using the NextSeq platform (Illumina, Inc.). Illumina reads were *de novo* assembled using CLC Genomics Workbench v.7 (QIAGEN). BLASTn and BLASTp were used to identify the presence of genes in assemblies and was manually curated using Artemis (Carver et al., 2008). The genome sequences were automatically annotated using the PGAP (NCBI Prokaryotic Genome Annotation Pipeline v.4.3). Sequences were further compared and aligned with GenBank data using BLAST^2^ and Geneious software (Biomatters). Prophages were identified using PHASTER ^3^. Online tools of Center for Genomic Epidemiology were used to determine *in silico* serotype, plasmid classification (plasmid-based replicon typing [PBRT]), and multilocus sequence typing using SerotypeFinder, PlasmidFinder, and MLST databases^4^. Genome sequence alignments and analysis were performed using Geneious (Biomatters) and Mauve (Darling et al., 2010). For core genome analysis, insertion elements, transposons and other mobile genetic elements and RNAs were excluded and concatenated sequences was used to infers phylogeny. Single-nucleotide polymorphisms (SNPs) calling and phylogeny were determined using CSI Phylogeny v.1.4 tool^5^. Genome of K12 MG1655 strain was used as reference genome. A final maximum-likelihood tree was constructed using Geneious (Biomatters).

### Nucleotide Sequence Accession Numbers

This Whole Genome Shotgun project has been deposited at DDBJ/ENA/GenBank under the accession PELA00000000 (CA14) and PEKZ00000000 (PA58). The version described in this paper is version PELA01000000 (CA14) and PEKZ01000000 (PA58).

## Results

The eight DEC strains, those were previous identified as *eae*, and *stx*2 positive, also harboring the *bfpA* gene. The strains belong to the O137:H6 serotype, sequence type (ST) 2678 and *stx*2 was subtyped as *stx*2f. The strains were also positive for *fimH* and *ibeA* by PCR, but negative for all other screened ExPEC virulence genes. In addition, all strains harbor genes encoding *yfaL*, an AIDA-I family autotransporter and the cytolethal distending toxin (*cdtB*) virulence determinants.

**FIGURE 1:**
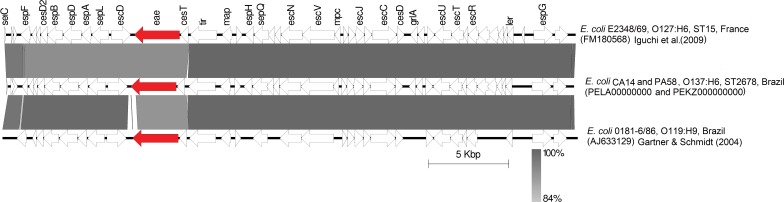
BLAST comparison of LEE region of CA14 and PA58 with reference strains. Coding regions are indicated by arrows pointing in the direction of transcription. Intimin (*eae*) and Stx-encoding regions are depicted in red.

Considering the presence of *eae* and *bfpA* as a genetic profile of tEPEC and the presence of *stx* gene as a genetic marker of STEC we investigated whether these strains were in fact tEPEC/STEC hybrids. Whole genome sequence of two strains (CA14 and PA58) confirmed the presence of *eae*, *bfpA*, and *stx2f*, as well as other virulence genes detected by PCR. The genetic environment of these three genes was the same in both strains (100% nucleotide identity). The locus of enterocyte effacement (LEE) region of both strains are 35,319 bp long with 96% of nucleotide identity (100% coverage) with the LEE region of prototype EPEC strain E2348/69 (O127:H6) (Figure 1; Gärtner and Schmidt, 2004; Iguchi et al., 2009). Through *in silico* analysis it was possible to verify that the two strains possess non-LEE encoded effectors EspJ, NleB, NleC, NleD, and NleH.

**FIGURE 2:**
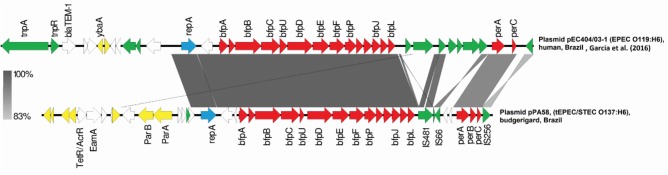
BLAST comparison of *bfp* plasmid of CA14 and PA58 with reference strains. Coding regions are indicated by arrows pointing in the direction of transcription. Plasmid replication genes are showed in blue, *bfp* and *per* operons are in red, insertion sequences related genes in green, and plasmid core genome genes are in yellow. Hypothetical proteins and other genes depicted in white.

The *bfp* genes were located in a ∼110 kb plasmid of incompatibility group FII-FIB, also was possible to determine the presence of this plasmid in all strains. Interestingly, using the PBRT technique, all strains were positive for the FIB replicon and negative for FII. In the analysis of the plasmids in the two genomes, it was possible to identify the FII replicon in addition to the FIB. However, in the FII replicon sequence it was possible to verify that the FII primers used in the PBRT technique have mismatches that prevent the amplification, resulting negative for this replicon. Blast searches showed that the *bfp/per* operons of pPA58 and pCA14 match with pEC404/03-1, a FIB plasmid from EPEC O119:H6 isolated from human in Brazil (Garcia et al., 2016). The alignment of 582 nucleotides of *bfp*/*per* operons with pEC404/03-1 is shown in Figure 2. *bfpA* genes from PA58 and CA14 have 97.8% of nucleotide identity with *bfpA* gene present in pMAR2, plasmid bearing *bfpA* gene from prototypic strain E2348/69. In both sequenced strains, the LEE region is integrated into the tRNA gene *selC*, as well as in the E2348/69 prototype tEPEC strain. The *bfp-pe*r operons are integrated into backbone region of the plasmids, close to the replication gene (*repA*). These genetic insertion contexts can be seen (Figures 1, 2). The size of the contig, in which, the subunits of the Stx2f toxin are present in the *de novo* assemblies, it was not possible to determine the genomic insertion site of the phage.

**FIGURE 3:**
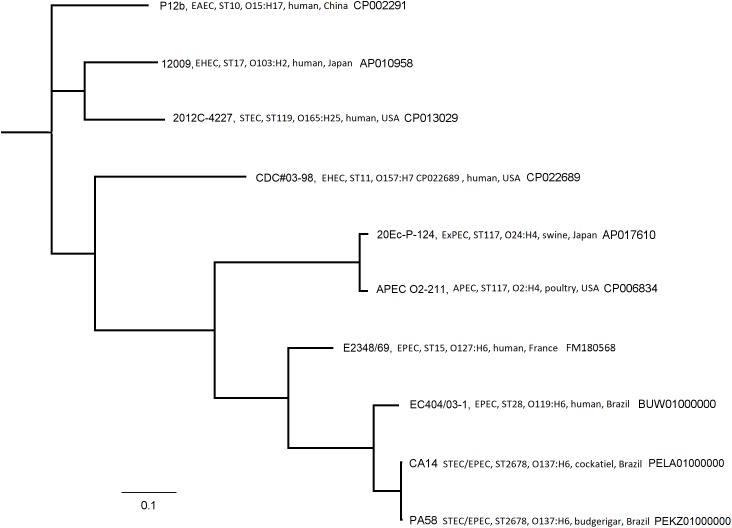
Phylogenetic tree based on a SNP-calling of core genome of PA58 and CA14 *Escherichia coli* strains and a selection of GenBank *E. coli* (EPEC, STEC, and other pathotypes) reference genomes retrieved from GenBank.

The PHASTER analysis shows that PA58 strain harbors 7 prophages, two of them complete; and strain CA14 harbors 9 prophages, two of them complete, as well. In both strains the *stx2f* was present in a prophage that has homology (70%) with a non-*stx* prophage present in the genome of an enterohemorrhagic *E. coli* (EHEC) strain O103:H2 isolated in Japan (Murakami et al., 2014). Moreover, the prophage here presented has low identity with Stx2f-encoding phages. Core genome SNP tree (Figure 3) grouped the genome of CA14 and PA58 in a clade with other typical EPEC strains (E2348/69 and EC404/03-1), suggesting that our strains are tEPEC that evolves integrating a prophage harboring Stx2f toxin.

Phenotypical characteristics of the eight-tEPEC/STEC strains are shown in Table 1. After 6 h of incubation with HeLa cells the following adherence patterns were observed: localized adherence-like (LA-L; strains C12, PA56L- and CA14), aggregative (AA; strain PA58) and undetermined (UND; strains C35, PA24, PA56L+ and PA08). The culture supernatants of all eight strains produced cytotoxic effect on Vero cells, and neutralization of the cytotoxic effect was observed in 6 out of the 8 strains using a polyclonal Stx2 antibody (Table 1). As positive control EDL933 was employed and showed 98% of cytotoxic effect, this effect was completely abolished by the Stx2 polyclonal antibody.

**Table 1:** Phenotypical characteristics of tEPEC/STEC strains.

Strain	Adherence pattern on HeLa cells^a^ (6-h assay)	Vero cytotoxicity assay^b^ (%)	Cytotoxicity Neutralization assay^c^ (%)	Rabbit ileal loop (analysis of the intestinal mucosa)		
				Histopathology	SEM^d^	TEM^e^
CA35	UND	68	100	Picnose of apical enterocytes of villus, with		A/E lesion +
CA12	LA-L	72	96	bacterial adherence to brush border, sloughed	Weak bacterial	A/E lesion +
PA24	UND	89	77	cells mixed to erythrocytes in lumen, mucus	adherence with	A/E lesion -
				and few to moderate number of heterocytes in lamina propria	microvilli damage	A/E lesion -
PA56L+	UND	73	–	Necrosis of mucosa, with thrombus in lamina		
PA56L-	LA-L	56	72	propria, bacteria adherence to necrotic mucosa	Bacterial adherence not	NT
				and erythrocytes in lumen	observed	NT
PA08	UND	71	98	Bacteria and erythrocytes in lumen		NT
CA14	LA-L	81	–	Picnose of apical enterocytes of villus, with		NT
PA58	AA	66	91	bacterial adherence to brush border, sloughed		
				cells mixed to erythrocytes in lumen mucus,		
				rare heterocytes in lamina propria		

*^a^UND, undetermined adherence pattern; LA-L, localized adherence-like pattern; AA, aggregative adherence pattern. ^b^Vero cytotoxicity assay after 72 h of incubation. ^c^Neutralization assay in Vero cells using polyclonal Stx2 antibody (1:400). ^d^SEM, scanning electron microscopy. ^e^TEM, transmission electron microscopy; A/E, attaching and effacing lesion (pedestal formation); NT, non-tested.*

**FIGURE 4:**
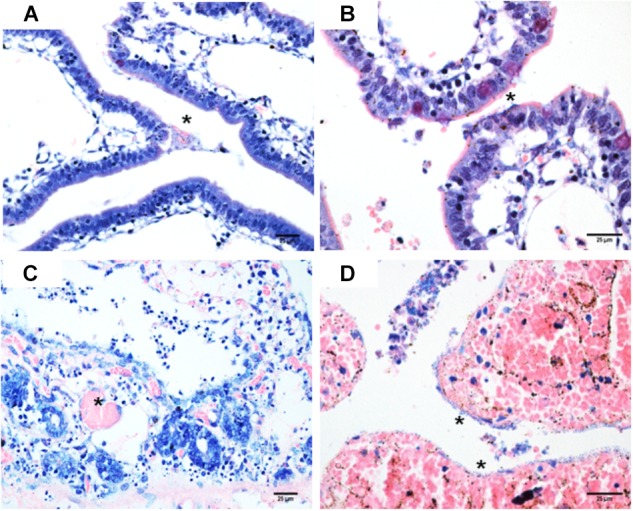
**(A)** Strain C14, picnose of apical enterocytes, bacterial and erythrocytes in intestinal lumen (^∗^). Giemsa, 400×; **(B)** Strain C35, bacterial in the brush border (^∗^), Giemsa, 400×; **(C)** Strain PA56L+, intestinal mucosa necrosis with thrombus in lamina propria (^∗^), Hematoxylin and eosin, 600×; **(D)** Strain PA56L–, necrosis, and hemorrhage of intestine mucosa with bacterial adherence (^∗^), Hematoxylin and eosin, 600×.

**FIGURE 5:**
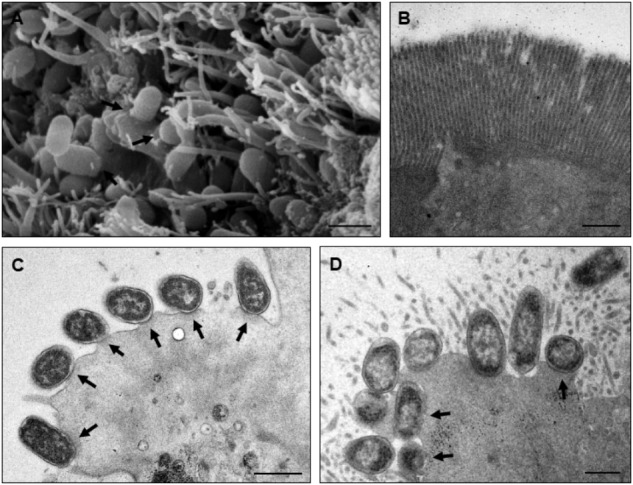
Rabbit ileal loop test analyzed by TEM and SEM. Panels **(A,C)** show strain PA24 adhered to the small intestine fragment forming pedestals (attaching and effacing lesion; indicated by arrows), respectively, by SEM and TEM. Reduction in the presence of microvilli can also be observed. **(B)** DH10-B and **(D)** E2348/69 were, respectively, used as negative and positive control strains for adherence and pedestal formation. Bars, 1 μm.

The macroscopic evaluation of the rabbit’s small intestine showed fluid accumulation with bloody mucus. Histopathological changes revealed sloughed cells and picnose (Figure 4A), bacterial adherence to brush border (Figure 4B), dilation of lymphatic vessels, necrosis of mucosa (Figures 4C,D) and heterophils in the lamina propria. Analysis by SEM (Figure 5A) showed that 4/8 strains presented bacterial adherence; while TEM confirmed the attaching and effacing (A/E) lesion in 2/4 adherent strains (Figures 5C,D). These results are presented on Table 1.

## Discussion

The pathotypes of DEC, a classification based on the presence or absence of virulence traits, was certainly valuable classification for epidemiological studies and diagnosis of diarrheas associated with *E. coli* infections. However, considering the genomic plasticity of *E. coli* and the current information obtained by whole genome sequencing, this classification is unable to group hybrid strains, resulting from the evolutionary process of EPEC (Ogura et al., 2009; Robins-Browne et al., 2016). Our study described eight hybrid tEPEC/STEC strains (*eae*^+^/*bfp*A^+^/*stx*2^+^) isolated from pet birds. These *stx2+* strains were previously investigated in a survey of DEC in companion birds (Gioia-Di Chiacchio et al., 2016) and grouped as a clade with 90–100% of similarity, by amplified fragment length polymorphism (AFLP). Here, these strains were submitted to genetic subtyping of the Stx-encoding gene, with positive result for *stx*2f gene. According to Murakami et al. (2014), human STEC Stx2f infection cases are becoming more frequent, with pigeons being considered as potential sources of this zoonosis (Murakami et al., 2014). De Rauw et al. (2018) screened a 27 years STEC, isolated in a university hospital in Belgium, and highlighted the emergence of STEC Stx2f, mainly in patients with HUS (De Rauw et al., 2018).

Shiga toxin production was observed in Vero cells and all strains were considered as highly cytotoxic, as all eight tEPEC/STEC supernatants killed more than 50% of Vero cells, when compared with the cell control. Despite the fact that, this polyclonal was raised against Stx2a it was able to neutralize the cytotoxicity effect of six Stx2f-producing strains, thus, the absence of neutralization in the strains PA56L+ and CA14 was probably due to high Stx secretion by them.

STEC Stx2f strains affecting pigeons and humans have been object of epidemiological investigations (Murakami et al., 2014; De Rauw et al., 2018). Studies revealed the presence of STEC Stx2f belonging to O15, O18ab:HNM, O25:H7, O45:HNM, O66:HNM, O75:HNM, O128:H2, O132, O135:HNM, and O152:HNM in Japanese and European pigeons (Murakami et al., 2014). In our study, all strains belonged to the O137:H6 serotype. According to the World Health Organization (WHO), O137:H41 serotype was reported as an agent of the HUS in humans (World Health Organization [WHO], 1998). O137:HNM were also detected in bovines in the State of Rio Grande do Sul, Brazil (Timm et al., 2007), but there is no evidence of the presence of this serotype in birds.

Pigeons from different countries were described as reservoir of *stx*2f+ *E. coli* belonging to distinct serotypes, and such strains mentioned as non-O157 EHEC have also been reported as a cause of disease in humans (World Health Organization [WHO], 1998; Schmidt et al., 2000). The isolation of STEC Stx2f in companion birds warns about the zoonotic risk, as these pets are closer and closer to humans. The importance of investigation in psittacine birds reinforces the need for studies involving these pathotypes and derivative EPEC hybrids.

Sanches et al. (2017) reported the isolation of tEPEC in psittacine birds (Sanches et al., 2017). It is well known that tEPEC is considered rare in animals, and that it is mainly found in humans (Hernandes et al., 2009). The presence of tEPEC in psittacidae suggests that a transmission of zooanthroponotic nature is more probable (Sanches et al., 2017). The strains of this study were characterized as tEPEC bearing an *stx* prophage. Also, the strains were highly cytotoxic *in vitro*, causing tissue damage in rabbit ileal loop model (Figure 4) and, some of them, induced A/E lesions *in vivo* (Figures 5C,D). This is an important finding, as there are no reports in the literature concerning these data.

Nyholm et al. (2015) stated that DEC could acquire virulence genes by horizontal transfer from other pathotypes; thereby leading to the development of pathotypes referred to as intermediaries, hybrids, or combined virulence (Nyholm et al., 2015). The emergence of new hybrid pathogens should be taken into consideration as for both patient care and epidemiological surveillance.

STEC/ETEC hybrid strains coding heat stable enterotoxin (ST) have been more frequently isolated (Leonard et al., 2016; Fatema-Tuz et al., 2017; Johura et al., 2017). STEC/ETEC strains isolated from clinical and environmental samples may represent emerging threatening pathogenic agents of food origin. The characterization of these strains is important in evaluating virulence potential, helping with the development of pathogen detection methods, and understanding how hybrid lineages evolve to have greater impact on public health.

MLST has been considered as a gold standard technique for evaluating pathogenic lineages of *E. coli*. In our study, the MLST results showed that all strains belonged to the sequence type ST2678. This ST was previously described (Wijetunge et al., 2015) as ESBL-positive O137:H6 serotype, isolated from human in United States (Wijetunge et al., 2015). The Enterobase Database also presented an EPEC strain ST2678 (serotype O137:H6), isolated from human in the United Kingdom, in 2016 (see text footnote 1). Interestingly, the *in silico* analysis classified the PA58 and CA14 strains to B2 phylogenetic group. In addition, these strains harbor *ibeA*, a gene that encodes the invasion brain endothelium protein, a virulence factor associated with neonatal meningitis *E. coli* (NMEC) and avian pathogenic *E. coli* (APEC) (Germon et al., 2005; Cunha et al., 2017). This fact may be associated with the phylogenetic background of these strains, since this virulence determinant is associated with phylogenetic B2 (Germon et al., 2005).

BLAST searches of the whole-genome sequences and comparative analysis with tEPEC strains E2348/69 (Iguchi et al., 2009) and O119:H6 (Garcia et al., 2016) point that PA58 and CA14 were more related with tEPEC genomes, and probably they became hybrids after the acquisition of *stx*-prophage. Our conclusions about evolutionary event of these hybrid strains agree with those observed (Hazen et al., 2017) in a phylogenomic study with hybrid EPEC/LT-producing ETEC isolates from children in Africa, which are genomically related to EPEC, but acquired ETEC virulence genes by horizontal via (Hazen et al., 2017).

Genomic analysis of two strains (CA14 and PA58) showed the location of *bfp* and *per* operons in a ∼110 kb plasmid belonging to the incompatibility group F (IncF) and possess FII-FIB replicons. In a search for complete sequences of plasmids harboring *bfp*, we verified that FII-FIB replicons are the most common plasmids in tEPEC. Plasmids of tEPEC pB171 (GenBank Accession No. AB024946) (Tobe et al., 1999), pMAR7 (GenBank Accession No. DQ388534) (Brinkley et al., 2006), and pMAR2 (GenBank Accession No. FM180569) (Iguchi et al., 2009) possesses FII-FIB replicons, as well as pPA58 and pCA14.

Figure 2 shows the blast alignments of the sequences of *bfp* and *per* regions, comparing our plasmids with sequences of E2348/69 (Iguchi et al., 2009), EC 0181-6/86 (Gärtner and Schmidt, 2004), and AJ633129 strains (Gärtner and Schmidt, 2004). In the draft genomes of PA58 and CA14, the contigs on which are present replication plasmid genes, *bfp* and *per* operons has high nucleotide identity with pEC404/03, an IncFIB plasmid from a tEPEC 0181-6/86 strain, serotype O119:H6 isolated in Brazil (Garcia et al., 2016). However, the backbone, region that includes genes involved in maintenance, conjugation and other core genes were different.

Our hybrid strains displayed three different adherence patterns on HeLa cells after 6 h of incubation, in the presence of mannose. Three strains presented undetermined and other three LA-L patterns. LA-L is characteristic of atypical EPEC resulting of BFP absence (Gomes et al., 2016), suggesting that BFP is not produced by these three strains. On the other hand, the PA58 hybrid strain displayed an AA pattern, but genes associated with EAEC adherence were not found in its genome indicating that another factor is involved in establishment of the this pattern. Further studies are necessary to decipher the adherence traits of these hybrids strains.

*In vivo* inoculation results evidenced the injury of enterocytes, as confirmed by histopathology. Autopsy revealed macroscopic lesions in the small intestine, with fluid accumulation in variable quantities and mucus with bloody appearance, while histopathological exams evidenced villus changes with desquamated necrotic cells, red blood cells and coccoid bacteria mixed with the mucus in the lumen. Dilated lymphatic vessels, as well as heterophils moderately infiltrated into the submucosa were also observed. These data corroborate the results presented (Pakpinyo et al., 2002) in naturally occurring cases of poultry enteritis-mortality syndrome associated with EPEC infection (Pakpinyo et al., 2002). Wales et al. (2005) state that histopathological descriptions of intestines colonized by bacteria producing A/E lesion usually adhere enterocytes in an extensive or multifocal pattern and typically have a distinct coccoid appearance (Wales et al., 2005). Colonized cells show to be degenerated and many are hyperchromatic, round, or picnotic. Detachment of enterocytes is commonly observed along with an inflammatory infiltrate of variable intensity with heterophils in the lamina propria.

Regarding SEM and TEM, it was possible to visualize bacteria adhered to enterocytes (4/8 strains), with destruction of microvilli, and pedestal formation (2/8 strains), thereby confirming its capacity to induce the A/E lesion *in vivo* (Figure 5). A/E lesion is the hallmark of EPEC/EHEC pathogenesis, and such characteristic of the studied isolates indicates their pathogenic potential, which corroborates the observations of other authors who used this model to confirm the pathogenic potential of human DEC lineages (Vieira et al., 2010; Piazza et al., 2013; Lewis et al., 2015).

Seeley et al. (2014) described an outbreak of enteritis in a population of captive parakeets living at the Massachusetts Zoo and studied *attaching-effacing E. coli* in captive budgerigars (Seeley et al., 2014). The authors stated that, unlike cattle, psittacine birds are generally not considered as EPEC reservoirs and pointed out the zoonotic importance of EPEC in the microbiota of some birds. There are few works evaluating the intestinal pathogenicity of STEC/EHEC or EPEC in birds.

The hybrid tEPEC/STEC strains found in this study reinforce the need of further studies, since this is an unpublished finding in both human and veterinarian literature, and represents an emerging threat as a bird pathogen, not to mention the potential consequences for public health. The phenotypic and genotypic characterization of DEC of bird origin may contribute to the diagnosis and adoption of preventive measures when breeding and keeping small psittacine birds in captivity.

## Conclusion

This work reports, for the first time, the presence of tEPEC/STEC hybrid strains in captive birds, and the evolutionary analysis suggests the acquisition of Stx2f-encoding phage by a tEPEC. The strains evaluated in this study belong to serotype O137:H6 (ST2678), were able to adhere to HeLa cells and produce toxins *in vitro*. They induced an intestinal inflammatory process and some strains promoted the attaching and effacing lesion *in vivo*. Together, these data confirm the pathogenic potential of strains present in pet birds.

## Article Summary

This article describes the emergence of hybrid strains of typical enteropathogenic *Escherichia coli* and Shiga-toxin producing *E. coli* in pet birds from Brazil. These strains (serotype O137:H6 and ST2678) were considered highly cytotoxic and capable of inducing attaching and effacing lesion *in vivo*, suggesting a potential public health concern.

## Author Contributions

RP and TK conceived and designed the research. RG-DC, MC, LMS, YD, CP, FM, DM, LFS, BG, MF, and CA performed the experiments. LFS, BG, WE, RP, and TK contributed with reagents, material, and analysis tools. RG-DC, MC, LMS, FM, CA, WE, RP, and TK analyzed the data. RG-DC, MC, CA, BG, WE, RP, and TK wrote the paper. All authors reviewed the manuscript and approved the final draft of the manuscript.

## Conflict of Interest Statement

The authors declare that the research was conducted in the absence of any commercial or financial relationships that could be construed as a potential conflict of interest.

## Footnotes

1 http://enterobase.warwick.ac.uk/species/index/ecoli

2 http://blast.ncbi.nlm.nih.gov/Blast.cgi

3 www.phaster.ca

4 https://cge.cbs.dtu.dk/

5 https://cge.cbs.dtu.dk/services/CSIPhylogeny
