# Supplementary material for: Novel Hybrid of Typical Enteropathogenic Escherichia coli and Shiga-Toxin-Producing E. coli (tEPEC/STEC) Emerging From Pet Birds
Source: Front Microbiol. 2018 Dec 6;9:2975. doi: 10.3389/fmicb.2018.02975 (PMC6291465; doi:10.3389/fmicb.2018.02975)
Supplement: Supplementary file 1 [file Table_1.docx]

**Table S1. Primer sequences and PCR conditions to study virulence genes**

| **Gene** | **Sequence (5´- 3´)** | **Annealing Temperature (°C) - incubation time** | **Fragment (pb)** | **Primers Reference** |
| --- | --- | --- | --- | --- |
| *bfpA* | GGTCTGTCTTTGATTGAATC | 55 -1 min | 485 | Munhoz et al., 2018 |
|  | TTTACATGCAGTTGCCGCTT |  |  |  |
|  |  |  |  |  |
| *eaeA* | CATTGATCAGGATTTTTCTGGT | 55 - 2 min | 510 | Mora et al., 2012 |
|  | TCCAGAATAATATTGTTATTACG |  |  |  |
|  |  |  |  |  |
| *stx*2a | GCGATACTGRGBACTGTGGCC | 64- 40 sec | 347 | Scheutz et al., 2012 |
|  | GCCACCTTCACTGTGAATGTG |  |  |  |
|  |  |  |  |  |
| *stx*2b | AAATATGAAGAAGATATTTGTAGCGGC | 64- 40 sec | 251 | Scheutz et al., 2012 |
|  | CAGCAAATCCTGAACCTGACG |  |  |  |
|  |  |  |  |  |
| *stx*2c | GAAAGTCACAGTTTTTATATACAACGG | 64- 40 sec | 177 | Scheutz et al., 2012 |
|  | GTACCGGCCACYTTTACTGTGAATGTA |  |  |  |
|  |  |  |  |  |
| *stx*2d | AAARTCACAGTCTTTATATACAACGGGTG | 64- 40 sec | 179 | Scheutz et al., 2012 |
|  | TTYCCGGCCACTTTTACTGTG |  |  |  |
|  |  |  |  |  |
| *stx*2e | CGGAGTATCGGGGAGAGGC | 64- 40 sec | 411 | Scheutz et al., 2012 |
|  | CTTCCTGACACCTTCACAGTAAAGGT |  |  |  |
|  |  |  |  |  |
| *stx*2f | TGGGCGTCATTCACTGGTTG | 64- 40 sec | 424 | Scheutz et al., 2012 |
|  | TAATGGCCGCCCTGTCTCC |  |  |  |
|  |  |  |  |  |
| *stx*2g | CACCGGGTAGTTATATTTCTGTGGATATC | 64- 40 sec | 573 | Scheutz et al., 2012 |
|  | GATGGCAATTCAGAATAACCGCT |  |  |  |
|  |  |  |  |  |
| *fimH* | GACGTCACCTGCCCTCCGGTA | 63 - 1 min | 508 | Hernandes et al., 2011 |
|  | TGCAGAACGGATAAGCCGTGG |  |  |  |
|  |  |  |  |  |
| *yfaL* | AGCGGCTACGTCAATATGGG | 60- 30 sec | 669 | This study |
|  | TTCCAGCGAGGCGATAATCC |  |  |  |
|  |  |  |  |  |
| *cdtB* | AACCAATAGTCGCCCACAGG | 60- 30 sec | 291 | This study |
|  | ATCATCCAGTTAGCGGCCTG |  |  |  |
|  |  |  |  |  |
| *papC* | TGATATCACGCAGTCAGTAGC | 54 – 30 sec | 501 | Janβen et al., 2001 |
|  | CCGGCCATATTCACATAA |  |  |  |
|  |  |  |  |  |
| *sfaD-E* | CTCCGGAGAACTGGGTGCATCTTAC | 50 - 40 sec | 410 | Le Bouguenec et al., 1992 |
|  | CGGAGGAGTAATTACAAACCTGGCA |  |  |  |
|  |  |  |  |  |
| *afaBC* | GCTGGGCAGCAAACTGATAACCTC | 61 – 30 sec | 750 | Yamamoto et al., 1995 |
|  | CATCAAGCTGTTTGTTCGTCCGCCG |  |  |  |
|  |  |  |  |  |
| *hlyA* | AACAAGGATAAGCACTGTTCTGGCT | 61 – 30 sec | 1177 | Yamamoto et al., 1995 |
|  | ACCATATAAGCGGTCATTCCCGTCA |  |  |  |
|  |  |  |  |  |
| *cnf*1 | AAGATGGAGTTTCCTATGCAGGAG | 61 – 30 sec | 498 | Yamamoto et al., 1995 |
|  | CATTCAGAGTCCTGCCCTCATTATT |  |  |  |
|  |  |  |  |  |
| *tsh* | GGGAAATGACCTGAATGCTGG | 61- 30 sec | 420 | Maurer et al., 1998 |
|  | CCGCTCATCAGTCAGTACCAC |  |  |  |
|  |  |  |  |  |
| *sat* | TGCTGGCTCTGGAGGAAC | 55 – 30 sec. | 667 | Ewers et al., 2004 |
|  | TTGAACATTCAGAGTACCGGG |  |  |  |
|  |  |  |  |  |
| *vat* | TCCTGGGACATAATGGTCAG | 55 – 30 sec. | 981 | Ewers et al., 2004 |
|  | GTGTCAGAACGGAATTGT |  |  |  |
|  |  |  |  |  |
| *iucD* | TACCGGATTGTCATATGCAGACCGT | 61 – 30 sec. | 602 | Yamamoto et al., 1995 |
|  | AATATCTTCCTCCAGTCCGGAGAAG |  |  |  |
|  |  |  |  |  |
| *iroN* | AATCCGGCAAAGAGACGAACCGCCT | 60 – 30 sec | 553 | Johnson et al., 2006 |
|  | GTTCGGGCAACCCCTGCTTTGACTTT |  |  |  |
|  |  |  |  |  |
| *iss* | GTGGCGAAAACTAGTAAAACAGC | 61 – 30 sec | 760 | Horne et al. 2000 |
|  | CGCCTCGGGGTGGATAA |  |  |  |
|  |  |  |  |  |
| *Irp2* | AAGGATTCGCTGTTACCGGAC | 61 – 30 sec | 281 | Schubert et al., 1998 |
|  | TCGTCGGGCAGCGTTTCTTCT |  |  |  |
|  |  |  |  |  |
| *fyuA* | GCGAC GGGAAGCGA TTTA | 60 – 30 sec | 780 | Schubert et al., 1998 |
|  | CGCAGTAGGCACGATGTTGTA |  |  |  |
|  |  |  |  |  |
| *usp* | ACATTCACGGCAAGCCTCAG | 60 – 30 sec | 440 | Bauer et al., 2002 |
|  | AGCGAGTTCCTGGTGAAAGC |  |  |  |
|  |  |  |  |  |
| *neuS* | TATAATTAGTAACCTGGGGC | 54 – 30 sec | 927 | Tsukamoto, 1997 |
|  | GGCGCTATTGAATAAGACTG |  |  |  |
|  |  |  |  |  |
| *ibeA* | TGGAACCCGCTCGTAATATAC | 59 – 30 sec | 342 | Ewers et al., 2004 |
|  | CTGCCTGTTCAAGCATTGCA |  |  |  |
|  |  |  |  |  |
| *cvi/cva* | TCCAAGCGGACCCCTTATAG | 60 – 30 sec | 598 | Ewers et al., 2004 |
|  | CGCAGCATAGTTCCATGCT |  |  |  |
|  |  |  |  |  |

**Cited references in Table S1**

Bauer, R.J., Zhang, L., Foxman, B., Siitonen, A., Jantunen, M.E., Saxen, H., Mars, C.F. (2001). Molecular epidemiology of 3 putative virulence gene for *Escherichia coli* urinary tract infection usp-iha and iroN. *The Journal of Infect. Dis.* 185, 1521-1524.

Ewers, C., Janβen, T., Kiebling, S., Philipp, H.C., Wieler, L. (2004). Molecular epidemiology of avian pathogenic *Escherichia coli* (APEC) isolated from colisepticemia in poultry. *Vet. Microbiol.* 104, 91-101.

[Hernandes, R.T](https://www.ncbi.nlm.nih.gov/pubmed/?term=Hernandes%20RT%5BAuthor%5D&cauthor=true&cauthor_uid=21926222)., [Velsko, I](https://www.ncbi.nlm.nih.gov/pubmed/?term=Velsko%20I%5BAuthor%5D&cauthor=true&cauthor_uid=21926222)., [Sampaio, S.C](https://www.ncbi.nlm.nih.gov/pubmed/?term=Sampaio%20SC%5BAuthor%5D&cauthor=true&cauthor_uid=21926222)., [Elias, W.P](https://www.ncbi.nlm.nih.gov/pubmed/?term=Elias%20WP%5BAuthor%5D&cauthor=true&cauthor_uid=21926222)., [Robins-Browne, R.M](https://www.ncbi.nlm.nih.gov/pubmed/?term=Robins-Browne%20RM%5BAuthor%5D&cauthor=true&cauthor_uid=21926222)., [Gomes, T.A](https://www.ncbi.nlm.nih.gov/pubmed/?term=Gomes%20TA%5BAuthor%5D&cauthor=true&cauthor_uid=21926222)., et al. (2011). Fimbrial adhesins produced by atypical enteropathogenic *Escherichia coli* strains. [*Appl Environ Microbiol*.](https://www.ncbi.nlm.nih.gov/pubmed/21926222) 77, 8391-9.

Horne, S.M., Pfaff-Macdonough, S.J., Giddings, C.W., Nolan, L.K. (2000). Cloning and sequencing of the *iss* gene from a virulent *Avian Escherichia coli.* Avian Dis. 44, 179-184.

Janβen, T., Scharz, C., Preikschat, P., Voss, M., Wieler, L.H. (2001). Virulence – associated genes in avian pathogenic *Escherichia coli* (APEC) isolated from internal organs of poultry having died from colibacillosis. *Int. J. Med. Microbiol.* 387, 371-378.

Johnson, T.J., Johnson, S. J., Nolan, L. K. (2006). Complete DNA sequence of a ColBM plasmid from avian pathogenic *Escherichia coli* suggests that it evolved from closely related ColV virulence plasmids. *J. Bacteriol.* 188, 5975-5983.

[Le Bouguenec, C](https://www.ncbi.nlm.nih.gov/pubmed/?term=Le%20Bouguenec%20C%5BAuthor%5D&cauthor=true&cauthor_uid=1349900)., [Archambaud, M](https://www.ncbi.nlm.nih.gov/pubmed/?term=Archambaud%20M%5BAuthor%5D&cauthor=true&cauthor_uid=1349900)., [Labigne, A](https://www.ncbi.nlm.nih.gov/pubmed/?term=Labigne%20A%5BAuthor%5D&cauthor=true&cauthor_uid=1349900). (1992). Rapid and specific detection of the *pap*, *afa*, and *sfa* adhesin-encoding operons in uropathogenic *Escherichia coli* strains by polymerase chain reaction. [*J. Clin. Microbiol*.](https://www.ncbi.nlm.nih.gov/pubmed/?term=Le+Bouguenec+et+al.%2C+1992) 30, 1189-93.

Maurer, J.J., Brown, T.P., Steffens, W.L., Thayer, S.G. (1998). The occurrence of ambient temperature regulated adhesins, curli and the temperature sensitive hemagglutinin Tsh among avian *Escherichia coli*. *Avian Dis.* 42, 106-118.

Mora, A., López, C., Dhabi, G., López-Beceiro, A.M., Fidalgo, L.E., Díaz, E.A., Martinez-Carrasco, C., Mamani, R., Herrera, A., Blanco, J.E., Blanco, M. Blanco, M. (2012). Seropathotypes, phylogroups, Stx subtypes and intimin types of Shiga toxin-producing Escherichia coli strains from wildlife animals with the same characteristics as human pathogenic isolates. *Appl. Environm. Microbiol.* AEM-07520.

Munhoz, D.D., Nara, J.M., Freitas, N.C., Moraes, C.T.P., Nunes, K.O., Yamamoto, B.B., Vasconcellos, F.M., Martínez-Laguna, Y., Girón, J.A., Martins, F.H., Abe, C.M., Elias, W.P., Piazza, R.M.F. [Distribution of Major Pilin Subunit Genes Among Atypical Enteropathogenic *Escherichia coli* and Influence of Growth Media on Expression of the *ecp* Operon.](https://www.ncbi.nlm.nih.gov/pubmed/29867850) *Front Microbiol*. 9:942. eCollection 2018.

Scheutz, F., Teel, A.L.D., Beutin, L., PiéRard, C.D., Buvens, D.G., Karch, D.H., et al. (2012). Multicenter evaluation of a sequence-based protocol for subtyping Shiga toxins and standardizing stx nomenclature. *J Clin. Microbiol*. 50, 2951–2963.

Schubert, S., Rakin, A., Karch, H., Carniel, E., Heesemann, J. (1998). Prevalence of the “High-Pathogenicity Island” of Yersinia Species among *Escherichia coli* Strains That Are Pathogenic to Humans. *Infect. Immun*. 66, 480-485.

Tsukamoto, T. (1997). PCR method for detection of K1 antigen and serotypes of *E. coli* isolated from extraintestinal infection. *Kansenshogaku Zasshi* 71, 125-129.

Yamamoto, S., Terai, A., Yuri, K., Kurazono, H., Takeda, Y., Yoshida, O. (1995). Detection of urovirulence factors in *Escherichia coli* by multiplex polymerase chain reaction. *FEMS Immun. Med. Microbiol.* 12, 85-90.
